# Origins and Evolution of the α-L-Fucosidases: From Bacteria to Metazoans

**DOI:** 10.3389/fmicb.2019.01756

**Published:** 2019-08-27

**Authors:** Jia You, Shujin Lin, Tao Jiang

**Affiliations:** ^1^Department of Hepatology, The Liver Center, The First Affiliated Hospital of Fujian Medical University, Fuzhou, China; ^2^College of Biological Science and Engineering, Fuzhou University, Fuzhou, China; ^3^Department of Urology, The First Affiliated Hospital of Fujian Medical University, Fuzhou, China

**Keywords:** α-L-fucosidase, evolution, sequence similarity network, bacteria, metazoa

## Abstract

α-L-fucosidases (EC 3.2.1.51, FUC), belonging to the glycoside hydrolase family 29 (GH29), play important roles in several biological processes and are markers used for detecting hepatocellular carcinoma. In this study, a protein sequence similarity network (SSN) was generated and a subsequent evolutionary analysis was performed to understand the enzymes comprehensively. The SSN indicated that the proteins in the FUC family are mainly present in bacteria, fungi, metazoans, plants, as well as in archaea, but less abundantly. The sequences in bacteria were found to be more diverse than those in other taxonomic groups. The SSN and a phylogenetic tree both supported that the proteins in the FUC family can be classified into 3 subfamilies. FUCs in each subfamily are under the pressure of negative selection. The enzymes from metazoans, fungi, and plants separated into the three subfamilies and shared high similarity with the bacterial homologs. The multiple sequence alignment results indicated that the amino acid residues for binding α-L-fucosidase and catalysis are highly conserved in the 3 subfamilies; however, the evolutionary patterns were different, based on the coevolution analysis in the subfamily of metazoans and bacteria. Finally, gene duplication plays an important role for α-L-fucosidase evolution, not only in metazoans, but also in bacteria and fungi.

## Introduction

Fucose is a hexose deoxy sugar with a relatively low abundance in the biosphere. Fucoses are usually attached to oligosaccharides, oligolipids, and other glycoconjugates by α-1,3 linkages to glucose (Glc), α-1,2 linkages to galactose (Gal), or 1,3/4/6 linkages to N-acetylglucosamine (GlcNAc) (Becker and Lowe, 2003). α-L-fucosidase (FUC) (EC 3.2.1.51) is a group of glycoside hydrolase (EC 3.2.1) that specifically catalyzes the reaction to remove the Non-reducing terminal L-fucose; recently, an FUC from the bacterium *Elizabethkingia meningoseptica* was found to have catalytic activity against the substrates with core α-1,3-fucosylation (Moreti et al., 2013; Li et al., 2018). FUC can be classified into two different glycoside hydrolase families: FUCs employing a retaining mechanism fall under the glycoside hydrolase family 29 (GH29), while those employing an inverting mechanism fall under the glycoside hydrolase family 95 (GH95) (Katayama et al., 2004). FUCs in the GH29 family are widespread in bacteria, fungi, molluscs, ascidians, and mammals (Intra et al., 2007). GH29 FUCs are well-studied because of the biological importance of L-fucose and the fucosylated conjugates in several critical biological processes such as the immune response, early embryogenesis and development, signal transduction, adhesion of pathogens, apoptosis, and extravasation of leukocytes (Intra et al., 2007). For example, fucose and FUC play important roles in gamete interactions in bull, ascidians, and molluscs (Matsumoto et al., 2002). FUC and/or L-Fucose may also have function in sperm-egg interaction in amphibians and mammals (Venditti et al., 2007).

In humans, FUC has great value for the diagnosis of hepatocellular carcinoma (HCC) as a serum marker (Gan et al., 2014; Wang et al., 2014). FUCs in humans are encoded by two genes: *hFUCA1*, which encodes the tissue enzyme, and *hFUCA2*, which encodes the plasma α-L-fucosidase. A deficiency of *hFUCA1* causes fucosidosis, a disease characterized by the excretion of Non-degraded oligosaccharides via urine and excessive deposition of oligosaccharides, mucopolysaccharides, and glycolipids in tissues (Willems et al., 1999). Fucosidosis patients have symptoms of neurodegeneration with progressive motor and mental deterioration. There are several studies that indicate a link between *hFUCA1* and tumorigenesis. For example, p53 can target hFUCA1 and regulate the growth and survival of cancer cells (Ezawa et al., 2016). hFUCA2 is a secreted enzyme that is fundamental for the adhesion of *Helicobacter pylori*, particularly the duodenal ulcer- and gastric cancer-specific strains (Liu T.W. et al., 2009), suggesting that hFUCA2 is a potential target for therapeutic intervention and clinical diagnosis of the diseases related to *H. pylori*.

FUCs in the GH29 family have been recognized widely in bacteria, ascidians, human, and other mammals. In the current research, sequence similarity networks (SSNs), multiple sequence alignments (MSAs), and other evolutionary analyses were carried out to obtain insights into the diversity and evolution of the FUC family across biospheres. We found that the FUC sequences were immensely more diverse than had been estimated previously, and that the proteins can be separated into 3 subfamilies.

## Materials and Methods

### Putative FUCs Sequences Collection

For a detailed analysis of FUCs across biospheres, the complete protein sequences of the FUCs that have been experimentally characterized were acquired from literature. These protein sequences were used as queries and putative FUCs were collected from UniProt. The *E*-value threshold is 10^−2^ during BLASTP. The Pfam database was further employed to identify the presence of the FUC domain (Marchler-Bauer et al., 2015). The protein sequences are listed in the Supplementary Sheet.

### MSAs and SSNs Analysis

MSAs of the putative FUC sequences were performed using the Multiple Alignment using Fast Fourier Transform (MAFFT) (version 7.0) program (Katoh et al., 2017). The SSNs of FUC sequences were created by the EFI-EST (Gerlt et al., 2015). Cytoscape was used to show the SSNs Shannon et al. (2003). The nodes in the networks indicate putative FUCs. An edge implies that the two putative FUCs linked by that edge show high sequence similarity Gerlt et al. (2015).

### Phylogenetic Analysis and Coevolving Protein Residues

MEGA7 was used to construct the unrooted phylogenetic trees using the maximum likelihood approach with 1000 bootstrap replicates (Kumar et al., 2016). The MISTIC web server was employed to calculate the coevolution in FUCs by the mutual information (MI) method in the MSAs (Simonetti et al., 2013). The three-dimensional structure of hFUCA1 was modeled using the I-TASSER server^1^ and visualized by PyMOL^2^.

### *K_a_*/*K_s_* Ratio Calculation

The Non-synonymous to synonymous substitution rate is indicated by *K_a_*/*K_s_*. PAL2NAL and was used to calculated the values (Suyama et al., 2006).

## Results

### Distribution of FUCs in Biospheres

**FIGURE 1:**
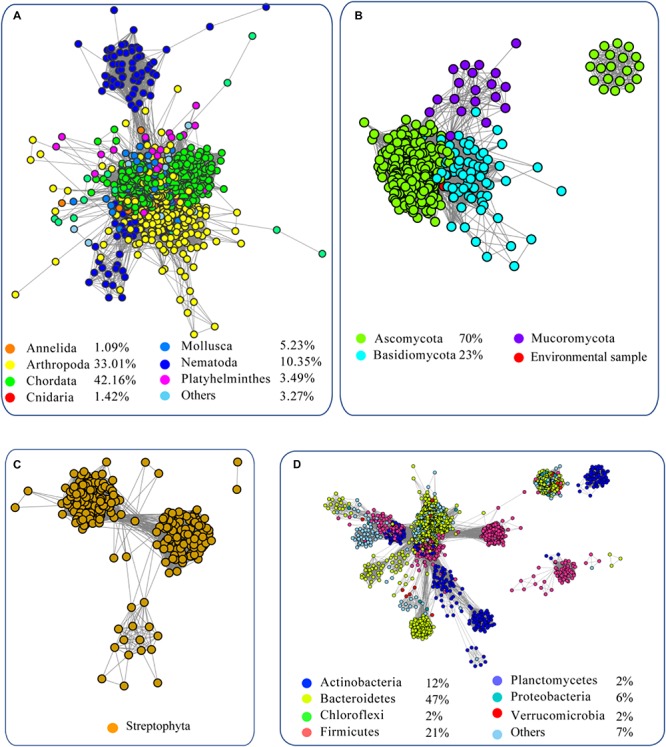
Sequence similarity network (SSN) of α-L-fucosidases from metazoans **(A)**, fungi **(B)**, plants **(C)**, and bacteria **(D)**. The sequences were retrieved from the UniProt database using BLASTP with an *E*-value = 10^–2^. The proteins with known function from the literature were used as query sequences. The SSN of the enzymes was constructed with an *E*-value = 10^–60^. Each node corresponds to one putative FUC. Edges are drawn with *E*-values < 10^–60^ for BLASTP. The colors represent different phyla and the percentage of the proteins in each phylum are recorded at the bottom.

To analyze the evolution of FUCs, we searched the UniProt database using the FUCs from humans (UniProt ID: P04066), *Arabidopsis* (Q8GW72), *Dictyostelium discoideum* (P10901), and *Bacteroides thetaiotaomicron* (Q9WYE2) as query sequences, which have been experimentally characterized. In total, 6208 sequences were collected (Supplementary Sheet) and FUC homologs were found to be widespread in metazoans, plant, fungi, and bacteria; in addition, a few homologs were also found in archaea. To provide a more comprehensive view of these sequences, the SSNs for the sequences from the domain Bacteria, kingdom Metazoa, clade Viridiplantae, and kingdom Fungi were constructed using a protein sequence similarity cut-off of 60% (Figure 1). The network was further illustrated by classifying the included taxa. In Metazoa, members of the FUC family were mainly found in Chordata, Arthropoda, Nematoda, Mollusca, and Platyhelminthes (Figure 1A). All of the homologs in Metazoa are clustered together, indicating that these enzymes are highly conserved. In fungi, FUCs can be found in the three common phyla: Ascomycota, Basidiomycota, and Mucoromycota. The proteins from Basidiomycota separated into two clusters at a protein sequence similarity cut-off is 60% (Figure 1B). In Viridiplantae, FUCs only occurred in Streptophyta, but not in the green algae such as Spirotaenia and Chlorophyta. The homologs in Streptophyta can be grouped into one cluster, suggesting that the sequences are also relatively conserved (Figure 1C). In Bacteria, homologs of FUCs mainly occurred in Actinobacteria, Bacteroidetes, Firmicutes, and Proteobacteria. The proteins in bacteria are relatively diverse because they separated into four clusters when the SSN was constructed using the same cut-off value (60% identity) (Figure 1D). In the taxonomic groups of Bacteria, Metazoa, Viridiplantae, and Fungi, FUCs were relatively low in Fungi. FUCs were encoded in *Aspergillus niger*, a model filamentous ascomycete fungus that is widely used in biotechnology. However, the gene cannot be found in *Neurospora crassa*, *Saccharomyces cerevisiae*, and other model fungi, implying that a loss of the gene *FUC* occurred widely in fungi. In conclusion, FUCs widely occurred in the biosphere and gene loss happened widely in fungi.

### Evolutionary Relationship of FUCs

**FIGURE 2:**
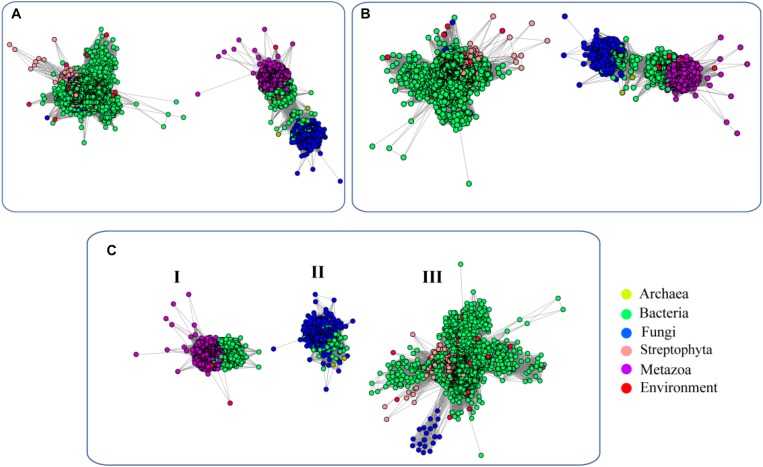
Sequence similarity network (SSN) of α-L-fucosidases across biospheres. The SSN of the putative FUCs was constructed with *E*-values = 10^–70^ **(A)**, 10^–75^ **(B)**, and 10^–80^ **(C)**. Each node corresponds to one putative FUC. Edges are required to have *E*-values less than the cutoff values. Metazoans, fungi, bacteria, and archaea proteins are displayed in purple, blue, green, and yellow, respectively. The putative FUCs from metagenomes are displayed in red.

To elucidate the evolutionary relationship of FUCs among the species, we performed an SSN analysis of the identified proteins. The results showed that the proteins from fungi and metazoans and some homologs in bacteria show similarity and fall into the same cluster at an *E*-value of 10^−70^ (protein sequence identity > 30%), but the proteins from plants and the bacterial homologs separated and formed another cluster (Figure 2A). If the *E*-value = 10^−75^ (protein sequence identity > 35%), the classification of these proteins shows a similar pattern as that if the *E*-value = 10^−70^ (Figure 2B). When the *E*-value cutoff = 10^−80^ (protein sequence identity > 40%), two clusters from metazoans and fungi separate (Figure 2C). In case of the two clusters, one cluster contains the proteins from bacteria and metazoans, and the proteins from the second cluster were from bacteria and fungi. The clusters containing the proteins from plant and bacteria cannot be separated at the cutoff value, suggesting that these enzymes are relatively highly conserved. In summary, the network analysis suggested that FUCs can be classified into 3 subfamilies, and FUCs from metazoans, fungi, and plants may share the common ancestors with the corresponding bacterial homologs.

**FIGURE 3:**
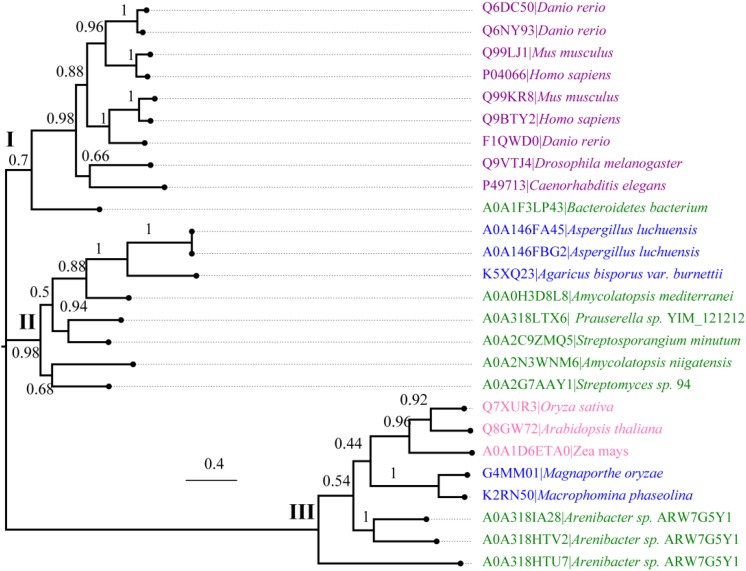
Phylogenetic analysis of α-L-fucosidases. Molecular phylogenetic analysis of the proteins generated via the maximum likelihood method using MEGA7. The replicate percentage of the trees is shown near the branches. The accession numbers of the proteins in the UniProt database are indicated in front of the names of the species. The proteins from metazoans, fungi, bacteria, and plants are highlighted in violet, blue, green, and pink.

**FIGURE 4:**
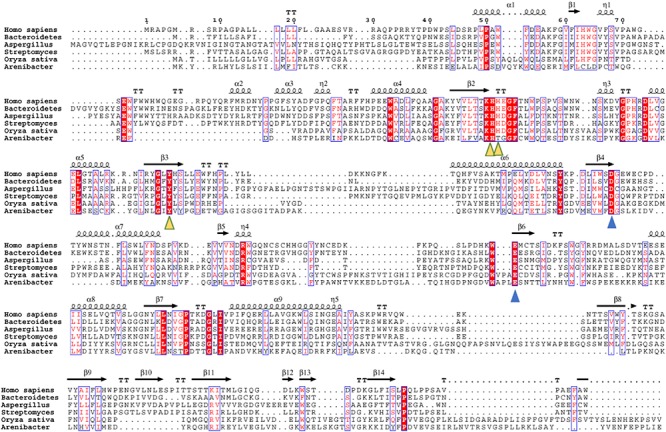
Multiple sequence alignment of the α-L-fucosidase homologs in the three subfamilies. Secondary structure elements (SSE) of hFUCA1 are shown at the top of the alignment. The amino acids that function as catalytic nucleophiles and the general acids/bases are blue triangles, and the residues that interact with fucose within the catalytic pocket are denoted as yellow triangles.

We further performed an evolutionary analysis using the representative proteins in each subfamily assigned based on SSNs to provide more detailed evolutionary relationships among the subfamilies (Figure 3 and Supplementary Figure S1). Proteins from subfamily I included the enzymes from humans, *Mus musculus*, *Danio rerio*, *Drosophila melanogaster*, and *Caenorhabditis elegans*. In human and *M. musculus*, there are two copies of each gene. In *D. rerio*, one copy of the gene was further duplicated, resulting in three copies. However, there is only one copy of *FUC* in *D. melanogaster* and in *C. elegans.* These proteins are clustered together with the enzyme from *B. thetaiotaomicron*, which has a known crystal structure (Wright et al., 2013). The proteins in subfamily II can be found in both fungi and bacteria. Generally, there is one copy of the gene in this subfamily, but the gene can also be duplicated in fungi, such as that in *Aspergillus luchuensis*. Most of the proteins in subfamily III are from bacteria and plants, while a few are from fungi. Similar to subfamily II, the gene in this subfamily can either be one or two copies because of gene duplication. In the genomes of *Oryza sativa* and *A. thaliana*, two model plant species, *FUC* from the GH29 family exists as a single copy. In this subfamily, the genes in some bacteria were duplicated, such as that in *Arenibacter* sp. A high bootstrap value supports the separation of these subfamilies in the phylogenetic tree. To further analyze the enzymes in the three subfamilies, the proteins from metazoans, fungi, and plants, as well as the corresponding enzymes in bacteria, were selected and aligned (Figure 4 and Supplementary Table S1). The enzymes from different subfamilies share conserved residues for catalysis and substrate binding (Figure 4). The enzymes in the same subfamily also shared relatively high identity in terms of amino acids sequences even though they are from different taxonomic groups (Supplementary Table S1). Based on SSN, phylogenetic tree, and sequence alignment, we suggested that the GH29 FUC family can be separated into 3 subfamilies, and that gene duplication occurred during evolution.

### Selection Pressure on FUCs

We measured the ratio of *dN*/*dS* of FUCs in the 3 groups with pairwise combinations of genes using PAL2NAL to analyze the selection pressure on the genes (Table 1 and Supplementary Figure S2). The results showed that the *dN/dS* ratios for the representative genes in the 3 subfamilies are <<1, indicating that the FUCs are under the pressure of negative selection. However, the ratios in the 3 subfamilies were slightly different: the ratios for *dN/dS* for the genes in metazoans are 0.16, while the values for the genes in other subfamilies are < 0.08. These results indicated that the FUCs in metazoans are subject to relaxed negative selection, while other subfamilies are under stringent negative selection.

**TABLE 1:** Tabular representation of the *dN/dS* calculations of α-L-fucosidases in the three subfamilies.

**Subfamily**	**UniProt ID**	**Number of Synonymous site (S)**	**Number of Non-synonymous site (N)**	**Synonymous substitution rate (dS)**	**Non-synonymous substitution rate (dN)**	**dN/dS**
I	Q9BTY2	320.6	1035.4	0.6313	0.1015	0.1608
II	A0A146FBG2	410.6	1311.4	9.3385	0.5992	0.0642
V	Q7XUR3	402.6	1103.4	4.6373	0.3412	0.0736

### Conservation and Evolution Analysis of the Amino Acid Residues in FUCs of Bacteria and Metazoans

**FIGURE 5:**
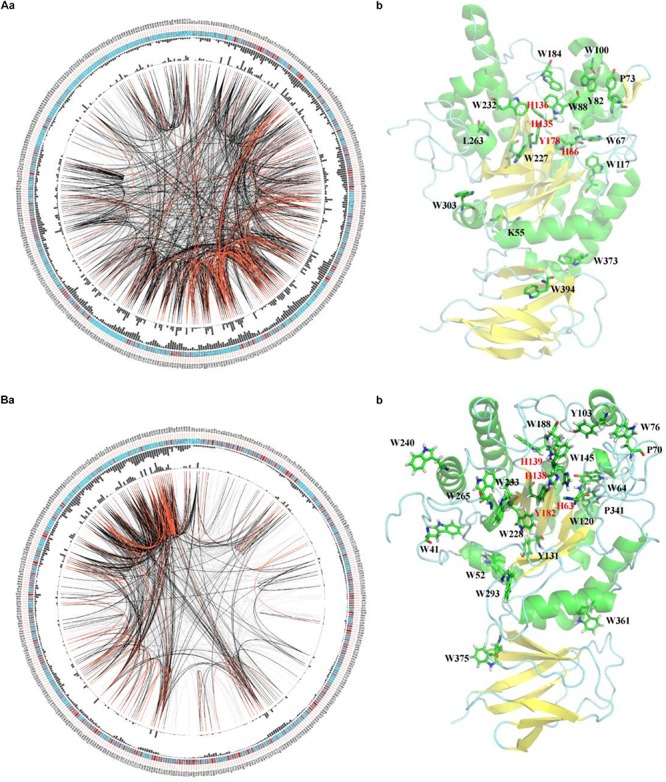
Conservation and coevolution of the amino acids in α-L-fucosidases represented by α-L-fucosidase from *B. thetaiotaomicron* **(A)** and human **(B)**. **(a)** Coevolution of the residues analyzed via the circular network. The connectivity of the coevolving residues is shown by the circular network. The labels in the outermost circle show the amino acid code and the alignment position of hFUCA1. In the second circle, the boxes indicate conservation in the multiple sequence alignment (MSA), from blue (less conserved ones) to red (highly conserved). The proximity and cumulative MI values as histograms are indicated in the third and fourth circles. The edges that link positions in the center of the circle correspond to a MI value higher than 6.5: red lines: top 5%, black lines: 70–95%, and gray lines: < 70%. **(b)** The top conserved FUC residues mapped in the structure of the proteins. The residues binding fucose within the catalytic pocket were highlighted in red.

To elucidate the similarity of FUCs between bacteria and metazoans, the sequences from the two groups were analyzed by MSAs. The sequences of hFUCA1 (UniProt ID: P04066) and FUC from *B. thetaiotaomicron* (PDB ID: 6hzy; named bFUC) were employed to display the results of MSA (Figure 5). To better illustrate the conservation of the amino acids, the 3D structure of hFUCA1 was constructed by the I-TASSER server (Figure 5). The MSA results showed that the residues in the N-termini are highly conserved compared to those in C-termini in both bacteria and metazoans. In the N-termini, the residues of hFUCA1 that bind fucose within the catalytic pocket include H63, H138, H139, and Y182, which is consistent with that found in a previous study (Liu S.W. et al., 2009). The corresponding amino acids in bFUC are H66, H135, H136, and Y178. The sequence WxDx that contains the catalytic nucleophile aspartate (D230 in hFUCA1 and D229 in bFUC) was also highly conserved. The D230N mutant lost catalytic activity significantly (Liu S.W. et al., 2009). Mapping the top conserved amino acids residues onto the structures indicated that the conserved amino acids that bind fucose and catalyze the reaction form a pocket in the N-terminus of the enzyme. Other conserved amino acid residues, including tryptophan, tyrosine, and proline, were located on the surface of the enzyme.

We further investigated the coevolution of the amino acids in FUCs using MI based on MSA (Figure 5A). Two residues are likely to be coevolving if they have a high MI score, which means that one amino acid mutation is related to another particular mutation in order to keep the function (Petit et al., 2014). The network of MI for the FUC members in bacteria indicated that the top 10% of the values were spread in the N-termini of the enzymes, while the values in the C-termini were relatively low (Figure 4A). However, the high values of the proteins in metazoans were in the C-termini. The analysis suggested that the evolutionary patterns of FUCs in bacteria and metazoans were different even though the conserved residues were highly similar.

## Discussion

FUCs, which are responsible for fucosylated glycoconjugate processing, are involved in cystic fibrosis, inflammation, lysosomal storage disease, cancer development, and in the interactions between gametes in both invertebrates and vertebrates. In this study, a comprehensive *in silico* study of FUCs was performed, which displayed that the homologs are distributed among bacteria, fungi, plants, and metazoans. Based on SSN and phylogenetic tree analyses, the proteins in the FUC family can be classified into 3 subfamilies and all of the subfamilies are under relaxed negative selection during evolution.

A previous study showed that FUCs were mainly from bacteria, fungi, and metazoans (Intra et al., 2007). In this study, we showed that FUCs also occurred in plants. In *Arabidopsis*, three enzymes (AtFuc95A, AtFUC1, and AtFXG1) were identified to have α-L-fucosidase activity (Frankova and Fry, 2013). Only AtFUC1 belongs to the GH29 family among the three enzymes (De La Torre et al., 2002), and we only considered the homologs of AtFUC1 in this study. The homologs from plants shared high sequence identity and showed high similarity to the bacterial homologues. Another previous study also showed that two copies of FUC genes are present in all vertebrates. Our research showed that one copy of the gene can be further duplicated, as the three copies of the gene encode the enzyme in *D. rerio*. In bacteria and fungi, the gene encoding FUC was also duplicated as two copies of the gene were found in many species.

The MSA with structure analysis showed that the conserved amino acids in FUCs can form a pocket in the N-termini for binding and catalyzing. The C-termini composed of β-sheets were less conserved. Several hydrophobic and large amino acids such as Trp and Tyr located near the surface of the enzyme were also conserved. The hydrophobicity of these amino acids may affect the solubility and function of the enzyme (Podorieszach and Huttunen-Hennelly, 2010), which in turn may also affect crystal growth important for structure determination. This may explain why the structures of hFUCA1 and hFUCA2 have not been solved until now. For the conserved amino acids, E289 may be responsible for general acid/base catalysis (Liu S.W. et al., 2009); however, according to the alignment, this amino acid is outside of the active site (Shaikh et al., 2013). Our study also showed that E289 is far from the active site and is less conserved. Cysteine was thought to be part of the active site, but cysteine was less conserved based on our analysis. These analyses suggested that the catalytic function of the amino acid residues in FUC is still unclear.

The FUC family can be divided into 3 subfamilies. Among them, the proteins from subfamily I were from bacteria and metazoans, the proteins from subfamily II were from bacteria and fungi, and the enzymes from subfamily III were from both bacteria and plants. This suggested that FUC appeared before bacteria and eukaryotes diverged. Furthermore, the results together with selection pressure analysis also suggested that FUCs were also conserved during evolution. Finally, we proposed that gene duplication is a common event in metazoans, as well as in bacteria and fungi during the evolution of FUC, as 2–3 copies of the gene can be found in the species.

## Data Availability

The raw data supporting the conclusions of this manuscript will be made available by the authors, without undue reservation, to any qualified researcher.

## Author Contributions

JY and TJ designed the experiments, performed all analysis, and wrote the manuscript. SL revised the manuscript and the data.

## Conflict of Interest Statement

The authors declare that the research was conducted in the absence of any commercial or financial relationships that could be construed as a potential conflict of interest.

## Footnotes

1 http://zhanglab.ccmb.med.umich.edu/I-TASSER/

2 https://pymol.org/
