# Supplementary material for: Origins and Evolution of the α-L-Fucosidases: From Bacteria to Metazoans
Source: Front Microbiol. 2019 Aug 27;10:1756. doi: 10.3389/fmicb.2019.01756 (PMC6718869; doi:10.3389/fmicb.2019.01756)
Supplement: Supplementary file 1 [file Image_1.pdf]

Supplementary Figure 1

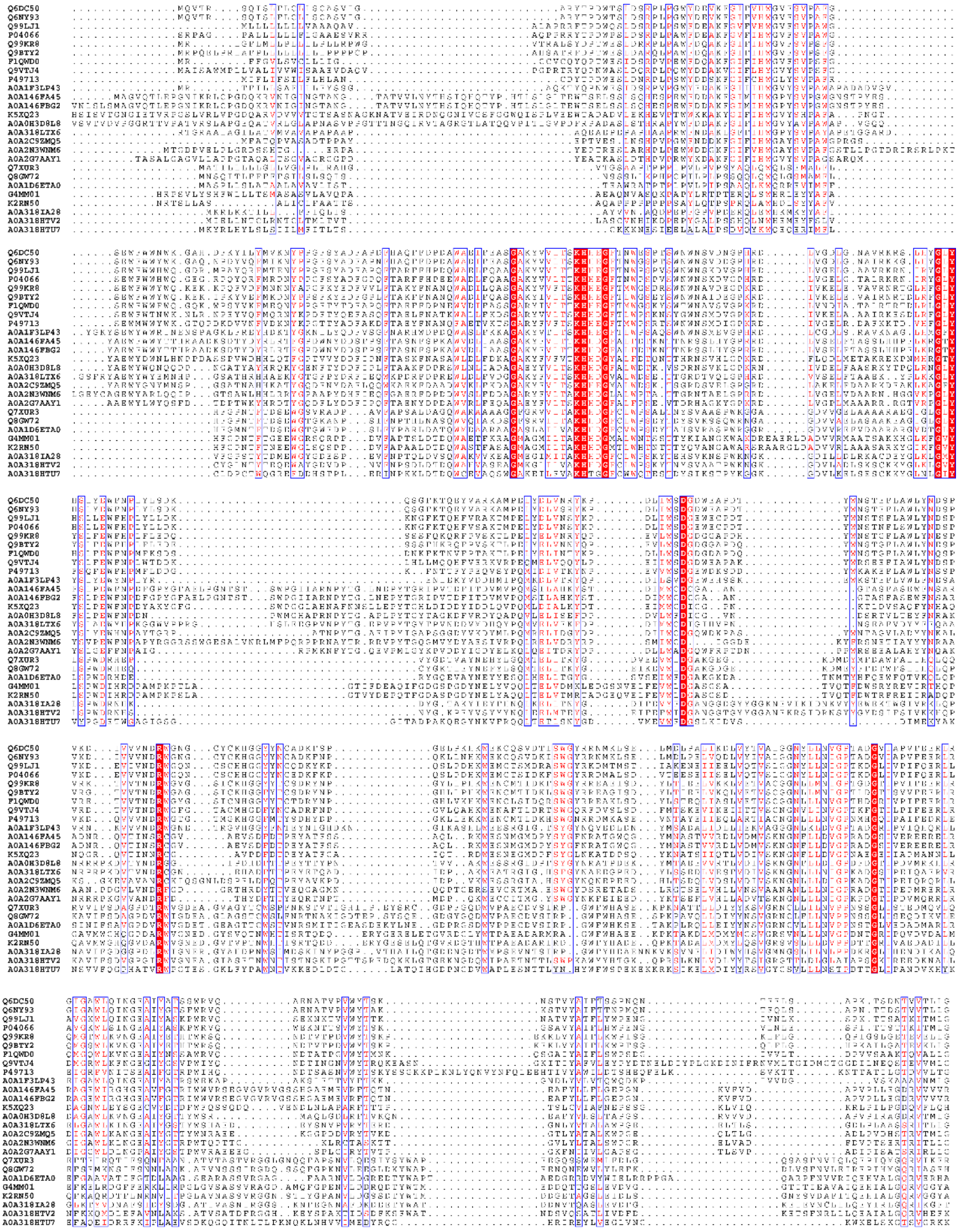

Supplementary Figure 1. **Multiple sequence alignment of the α- L-fucosidase homologs in the three subfamilies.** The sequences are corresponding in Figure 3.

Supplementary Figure 2

A

```
                          M   R   P   Q   E   L   P   R   L   A   F   P   L   L   L   L   L   L   L   L
sp|Q9BTY2|FUCO2_HUMAN     ATG CGG CCC CAG GAG CTC CCC AGG CTC GCG TTC CCG TTG CTG CTG TTG CTG TTG CTG CTG
                          -   -   -   -   -   -   M   R   L   G   F   L   M   L   L   P   L   L   L   L
sp|Q99KR8|FUCO2_MOUSE     --- --- --- --- --- --- ATG CGC TTG GGG TTC CTG ATG CTT CTG CCG CTG CTG CTG CTG

                          L   P   P   P   P   C   P   A   H   S   A   T   R   F   D   P   T   W   E   S
sp|Q9BTY2|FUCO2_HUMAN     CTG CCG CCG CCG CCG TGC CCT GCC CAC AGC GCC ACG CGC TTC GAC CCC ACC TGG GAG TCC
                          P   L   L   R   P   W   G   V   T   R   A   L   S   Y   D   P   T   W   E   S
sp|Q99KR8|FUCO2_MOUSE     CCG CTC CTG AGG CCT TGG GGG GTC ACG AGA GCC CTT AGC TAT GAC CCC ACT TGG GAA TCT

                          L   D   A   R   Q   L   P   A   W   F   D   Q   A   K   F   G   I   F   I   H
sp|Q9BTY2|FUCO2_HUMAN     CTG GAC GCC CGC CAG CTG CCC GCG TGG TTT GAC CAG GCC AAG TTC GGC ATC TTC ATC CAC
                          L   D   R   R   P   L   P   A   W   F   D   Q   A   K   F   G   I   F   I   H
sp|Q99KR8|FUCO2_MOUSE     CTG GAC AGA CGC CCG CTG CCA GCC TGG TTC GAC CAG GCC AAG TTC GGC ATC TTT ATC CAC

                          W   G   V   F   S   V   P   S   F   G   S   E   W   F   W   W   Y   W   Q   K
sp|Q9BTY2|FUCO2_HUMAN     TGG GGA GTG TTT TCC GTG CCC AGC TTC GGT AGC GAG TGG TTC TGG TGG TAT TGG CAA AAG
                          W   G   V   F   S   V   P   S   F   G   S   E   W   F   W   W   Y   W   Q   K
sp|Q99KR8|FUCO2_MOUSE     TGG GGA GTG TTC TCC GTG CCC AGC TTT GGT AGT GAA TGG TTC TGG TGG TAT TGG CAA AAG

                          E   K   I   P   K   Y   V   E   F   M   K   D   N   Y   P   P   S   F   K   Y
sp|Q9BTY2|FUCO2_HUMAN     GAA AAG ATA CCG AAG TAT GTG GAA TTT ATG AAA GAT AAT TAC CCT CCT AGT TTC AAA TAT
                          E   K   K   P   Q   F   V   D   F   M   N   N   N   Y   A   P   G   F   K   Y
sp|Q99KR8|FUCO2_MOUSE     GAG AAG AAA CCC CAG TTT GTG GAC TTT ATG AAC AAT AAT TAT GCT CCT GGT TTT AAG TAT

                          E   D   F   G   P   L   F   T   A   K   F   F   N   A   N   Q   W   A   D   I
sp|Q9BTY2|FUCO2_HUMAN     GAA GAT TTT GGA CCA CTA TTT ACA GCA AAA TTT TTT AAT GCC AAC CAG TGG GCA GAT ATT
                          E   D   F   V   V   L   F   T   A   K   Y   F   N   A   N   Q   W   A   D   I
sp|Q99KR8|FUCO2_MOUSE     GAA GAT TTT GTA GTG CTA TTT ACA GCC AAG TAT TTT AAT GCA AAC CAG TGG GCA GAT ATT

                          F   Q   A   S   G   A   K   Y   I   V   L   T   S   K   H   H   E   G   F   T
sp|Q9BTY2|FUCO2_HUMAN     TTT CAG GCC TCT GGT GCC AAA TAC ATT GTC TTA ACT TCC AAA CAT CAT GAA GGC TTT ACC
                          L   Q   A   S   G   A   K   Y   V   V   F   T   S   K   H   H   E   G   F   T
sp|Q99KR8|FUCO2_MOUSE     CTC CAG GCC TCT GGT GCC AAA TAT GTG GTC TTC ACT TCT AAA CAT CAC GAA GGC TTT ACA

                          L   W   G   S   E   Y   S   W   N   W   N   A   I   D   E   G   P   K   R   D
sp|Q9BTY2|FUCO2_HUMAN     TTG TGG GGG TCA GAA TAT TCG TGG AAC TGG AAT GCC ATA GAT GAG GGG CCC AAG AGG GAC
                          M   W   G   S   D   R   S   W   N   W   N   A   V   D   E   G   P   K   R   D
sp|Q99KR8|FUCO2_MOUSE     ATG TGG GGC TCA GAC CGT TCT TGG AAC TGG AAT GCA GTC GAT GAG GGG CCA AAG AGG GAC

                          I   V   K   E   L   E   V   A   I   R   N   R   T   D   L   R   F   G   L   Y
sp|Q9BTY2|FUCO2_HUMAN     ATT GTC AAG GAA CTT GAG GTA GCC ATT AGG AAC AGA ACT GAC CTG CGT TTT GGA CTG TAC
                          I   V   K   E   L   E   V   A   V   R   N   R   T   G   L   H   F   G   L   Y
sp|Q99KR8|FUCO2_MOUSE     ATT GTC AAG GAG CTT GAG GTG GCT GTG AGG AAC AGG ACT GGC TTG CAC TTT GGT CTG TAC

                          Y   S   L   F   E   W   F   H   P   L   F   L   E   D   E   S   S   S   F   H
sp|Q9BTY2|FUCO2_HUMAN     TAT TCC CTT TTT GAA TGG TTT CAT CCG CTC TTC CTT GAG GAT GAA TCC AGT TCA TTC CAT
                          Y   S   L   F   E   W   F   H   P   L   F   L   E   D   Q   S   S   S   F   Q
sp|Q99KR8|FUCO2_MOUSE     TAT TCT CTT TTT GAA TGG TTC CAT CCG CTC TTT CTG GAG GAT CAG TCC AGC TCA TTC CAA

                          K   R   Q   F   P   V   S   K   T   L   P   E   L   Y   E   L   V   N   N   Y
sp|Q9BTY2|FUCO2_HUMAN     AAG CGG CAA TTT CCA GTT TCT AAG ACA TTG CCA GAG CTC TAT GAG TTA GTG AAC AAC TAT
                          K   Q   R   F   P   V   S   K   T   L   P   E   L   Y   E   L   V   N   R   Y
sp|Q99KR8|FUCO2_MOUSE     AAG CAG CGA TTT CCC GTT TCT AAG ACA TTG CCT GAG CTC TAT GAG TTG GTG AAC AGA TAC

                          Q   P   E   V   L   W   S   D   G   D   G   G   A   P   D   Q   Y   W   N   S
sp|Q9BTY2|FUCO2_HUMAN     CAG CCT GAG GTT CTG TGG TCG GAT GGT GAC GGA GGA GCA CCG GAT CAA TAC TGG AAC AGC
                          Q   P   E   V   L   W   S   D   G   D   G   G   A   P   D   H   Y   W   N   S
sp|Q99KR8|FUCO2_MOUSE     CAG CCT GAA GTC CTG TGG TCA GAC GGA GAT GGG GGC GCG CCA GAT CAC TAC TGG AAC AGC
```

B

```
A0A146FBG2   ACA CTG GAG CCC GGA AAC ATC AAG CGA CTC TGC CCC GGC GAC CAA AAA CGC GTC AAC ATT
              T   L   E   P   G   N   I   K   R   L   C   P   G   D   Q   K   R   V   N   I
A0A146FA45   ACA CTG GAG CCC GGA AAC ATC AAG CGA CTC TGC CCC GGC GAC CAA AAA CGC GTC AAC ATT

              G   I   N   G   T   A   N   G   T   A   T   V   V   L   N   Y   T   H   S   I
A0A146FBG2   GGC ATC AAT GGT ACA GCC AAC GGA ACA GCA ACC GTC GTC CTG AAC TAC ACC CAC TCC ATC
              G   I   N   G   T   A   N   G   T   A   T   V   V   L   N   Y   T   H   S   I
A0A146FA45   GGC ATC AAT GGT ACA GCC AAC GGA ACA GCA ACC GTC GTC CTG AAC TAC ACC CAC TCC ATC

              Q   H   Q   T   Y   P   H   T   L   S   L   G   L   T   E   W   T   S   E   L
A0A146FBG2   CAG CAT CAA ACC TAC CCT CAC ACC CTC TCC CTC GGC CTA ACA GAA TGG ACC TCG GAG CTA
              Q   H   Q   T   Y   P   H   T   L   S   L   G   L   T   E   W   T   S   E   L
A0A146FA45   CAG CAT CAA ACC TAC CCT CAC ACC CTC TCC CTC GGC CTA ACA GAA TGG ACC TCG GAG CTA

              S   S   L   S   Q   H   E   S   P   E   W   F   D   D   A   K   F   G   I   M
A0A146FBG2   TCC AGC CTC TCG CAA CAC GAA AGC CCC GAA TGG TTC GAC GAC GCC AAA TTC GGC ATC ATG
              S   S   L   S   Q   H   E   S   P   E   W   F   D   D   A   K   F   G   I   M
A0A146FA45   TCC AGC CTC TCG CAA CAC GAA AGC CCC GAA TGG TTC GAC GAC GCC AAA TTC GGC ATC ATG

              I   H   W   G   P   Y   S   V   P   G   W   G   N   S   T   P   Y   E   S   Y
A0A146FBG2   ATC CAC TGG GGT CCT TAC TCC GTC CCC GGC TGG GGC AAC TCG ACA CCC TAT GAA AGC TAC
              I   H   W   G   P   Y   S   V   P   G   W   G   N   S   T   P   Y   E   S   Y
A0A146FA45   ATC CAC TGG GGT CCT TAC TCC GTC CCC GGC TGG GGC AAC TCG ACA CCC TAT GAA AGC TAC

              A   E   W   F   W   W   Y   T   T   H   R   A   A   D   K   S   D   T   Y   D
A0A146FBG2   GCC GAG TGG TTC TGG TGG TAC ACC ACG CAC CGC GCC GCC GAC AAA TCC GAC ACC TAC GAC
              A   E   W   F   W   W   Y   T   T   H   R   A   A   D   K   S   D   T   Y   D
A0A146FA45   GCC GAG TGG TTC TGG TGG TAC ACC ACG CAC CGC GCC GCC GAC AAA TCC GAC ACC TAC GAC

              Y   R   L   R   T   F   G   P   D   W   N   Y   D   D   S   F   P   S   F   T
A0A146FBG2   TAC CGA CTC CGC ACC TTC GGC CCT GAC TGG AAC TAC GAC GAC TCG TTC CCC TCC TTC ACG
              Y   R   L   R   T   F   G   P   D   W   N   Y   D   D   S   F   P   S   F   T
A0A146FA45   TAC CGA CTC CGC ACC TTC GGC CCT GAC TGG AAC TAC GAC GAC TCG TTC CCC TCC TTC ACG

              A   S   N   F   S   P   K   A   W   V   D   L   I   A   A   S   G   A   K   Y
A0A146FBG2   GCC TCC AAT TTC TCC CCC AAA GCC TGG GTG GAC CTA ATC GCG GCC TCC GGC GCC AAA TAC
              A   S   N   F   S   P   K   A   W   V   D   L   I   A   A   S   G   A   K   Y
A0A146FA45   GCC TCC AAT TTC TCC CCC AAA GCC TGG GTG GAC CTA ATC GCG GCC TCC GGC GCC AAA TAC

              F   V   L   T   T   K   H   H   D   G   F   A   L   F   D   T   K   N   T   T
A0A146FBG2   TTC GTA CTA ACA ACA AAA CAC CAC GAC GGC TTC GCG CTC TTC GAC ACC AAA AAC ACC ACG
              F   V   L   T   T   K   H   H   D   G   F   A   L   F   D   T   K   N   T   T
A0A146FA45   TTC GTA CTA ACA ACA AAA CAC CAC GAC GGC TTC GCG CTC TTC GAC ACC AAA AAC ACC ACG

              N   R   S   S   L   H   Y   G   P   R   R   D   L   V   S   E   L   F   T   A
A0A146FBG2   AAC CGC TCC TCC CTC CAC TAC GGC CCC CGC CGA GAC CTC GTG TCG GAA CTC TTC ACC GCA
              N   R   S   S   L   H   Y   G   P   R   R   D   L   V   S   E   L   F   T   A
A0A146FA45   AAC CGC TCC TCC CTC CAC TAC GGC CCC CGC CGA GAC CTC GTG TCG GAA CTC TTC ACC GCA

              S   S   L   H   H   P   F   L   K   R   G   T   Y   F   S   L   P   E   W   F
A0A146FBG2   TCC TCC CTC CAC CAC CCC TTC CTA AAA CGC GGC ACC TAC TTC TCC CTC CCC GAA TGG TTC
              S   S   L   H   H   P   F   L   K   R   G   T   Y   F   S   L   P   E   W   F
A0A146FA45   TCC TCC CTC CAC CAC CCC TTC CTA AAA CGC GGC ACC TAC TTC TCC CTC CCC GAA TGG TTC

              N   P   D   F   G   P   Y   G   F   A   E   L   P   G   N   T   S   T   S   W
A0A146FBG2   AAT CCA GAC TTC GGG CCC TAC GGG TTC GCC GAA CTA CCC GGT AAC ACC TCA ACG AGC TGG
              N   P   D   F   G   P   Y   G   F   A   E   L   P   G   N   T   S   T   S   W
A0A146FA45   AAT CCA GAC TTC GGG CCC TAC GGG TTC GCC GAA CTA CCC GGT AAC ACC TCA ACG AGC TGG

              P   G   I   I   A   R   N   P   Y   T   G   L   N   E   P   Y   T   G   R   I
A0A146FBG2   CCG GGC ATC ATC GCG CGC AAC CCC TAC ACG GGG CTC AAC GAG CCC TAC ACG GGC CGC ATC
              P   G   I   I   A   R   N   P   Y   T   G   L   N   E   P   Y   T   G   R   I
A0A146FA45   CCG GGC ATC ATC GCG CGC AAC CCC TAC ACG GGG CTC AAC GAG CCC TAC ACG GGC CGC ATC

              P   V   T   D   F   I   T   D   V   M   V   P   Q   M   S   I   L   A   H   K
A0A146FBG2   CCG GTC ACC GAC TTC ATC ACC GAC GTA ATG GTG CCC CAA ATG AGC ATC CTC GCA CAC AAG
              P   V   T   D   F   I   T   D   V   M   V   P   Q   M   S   I   L   A   H   K
A0A146FA45   CCG GTC ACC GAC TTC ATC ACC GAC GTA ATG GTG CCC CAA ATG AGC ATC CTC GCA CAC AAG
```

C

```
           M   A   T   I   L   L   L   L   L   G   L   L   V   G   L   P   L   L   R   A
Q7XUR3     ATG GCG ACC ATT CTT CTT CTT CTT CTT GGG TTG CTT GTC GGC CTC CCC CTC CTC CGC GCC
           M   N   S   Q   I   T   L   F   F   F   F   F   S   I   L   S   L   S   Q   I
Q8GW72     ATG AAC TCT CAA ATC ACT CTT TTC TTC TTC TTC TTC TCA ATC CTC TCC CTC TCC CAA ATC

           H   G   V   T   G   S   A   A   P   T   P   P   P   L   P   V   L   P   V   P
Q7XUR3     CAT GGC GTC ACC GGC TCC GCG GCC CCG ACG CCG CCG CCT CTG CCG GTC CTC CCC GTC CCC
           S   -   -   N   S   S   S   L   L   K   P   H   P   C   P   I   L   P   L   P
Q8GW72     TCA --- --- AAT TCA TCA TCA CTA CTA AAA CCA CAC CCA TGT CCA ATT TTA CCC CTC CCA

           S   Y   A   Q   L   Q   W   Q   L   S   E   M   A   L   F   L   H   F   G   P
Q7XUR3     TCC TAC GCG CAG CTC CAG TGG CAG CTC TCC GAG ATG GCC CTC TTC CTC CAC TTC GGC CCC
           S   S   Q   Q   L   Q   W   Q   L   G   S   M   A   M   F   L   H   F   G   P
Q8GW72     TCC TCC CAA CAG CTC CAA TGG CAA CTC GGA TCC ATG GCC ATG TTC CTC CAT TTT GGA CCC

           N   T   F   T   D   S   E   W   G   S   V   R   A   D   P   A   V   F   A   P
Q7XUR3     AAC ACC TTC ACG GAT TCC GAG TGG GGC TCC GTC CGC GCC GAC CCG GCC GTG TTC GCG CCC
           N   T   F   T   D   S   E   W   G   T   G   K   A   N   P   S   I   F   N   P
Q8GW72     AAC ACT TTC ACC GAC TCC GAA TGG GGC ACC GGA AAA GCT AAC CCA TCC ATT TTC AAC CCG

           S   A   L   D   A   G   Q   W   A   R   A   A   A   A   G   G   F   G   R   V
Q7XUR3     TCC GCG CTC GAC GCG GGC CAG TGG GCG CGC GCG GCG GCG GCG GGA GGG TTC GGG CGC GTC
           T   H   L   N   A   S   Q   W   V   Q   I   A   K   D   S   G   F   S   R   V
Q8GW72     ACC CAT CTC AAC GCA AGT CAA TGG GTC CAA ATC GCT AAA GAC TCA GGC TTT TCC CGC GTT

           V   L   T   A   K   H   H   D   G   F   C   L   W   P   S   A   L   T   N   Y
Q7XUR3     GTG CTC ACG GCC AAG CAC CAC GAC GGC TTC TGC CTC TGG CCC TCG GCC CTG ACG AAC TAC
           I   L   T   A   K   H   H   D   G   F   C   L   W   P   S   E   Y   T   D   Y
Q8GW72     ATC CTC ACA GCT AAG CAC CAT GAT GGA TTC TGT CTC TGG CCT AGC GAA TAC ACT GAT TAC

           S   V   A   A   S   P   W   K   G   G   A   G   D   V   V   G   E   L   A   A
Q7XUR3     TCG GTG GCC GCC TCT CCG TGG AAG GGC GGC GCC GGC GAC GTC GTC GGC GAG CTC GCC GCC
           S   V   K   S   S   Q   W   R   N   G   A   G   D   V   V   A   E   L   A   S
Q8GW72     TCC GTC AAA TCT AGC CAA TGG AGA AAC GGA GCC GGC GAT GTA GTG GCT GAA CTA GCT TCT

           A   A   R   A   E   G   I   G   L   G   L   Y   L   S   P   W   D   R   H   E
Q7XUR3     GCC GCG CGC GCC GAA GGT ATC GGG CTG GGG CTG TAC CTC TCG CCG TGG GAC CGG CAC GAG
           A   A   K   E   A   G   I   G   L   G   L   Y   L   S   P   W   D   R   H   E
Q8GW72     GCG GCA AAG GAA GCT GGA ATT GGA CTT GGT CTT TAC CTT TCT CCA TGG GAT CGA CAT GAA

           P   V   Y   G   D   T   V   A   Y   N   E   H   Y   L   G   Q   M   T   E   L
Q7XUR3     CCG GTG TAC GGC GAC ACC GTC GCG TAC AAC GAG CAC TAC TTG GGC CAG ATG ACG GAA TTG
           Q   C   Y   G   K   T   L   E   Y   N   E   F   Y   L   S   Q   M   T   E   L
Q8GW72     CAG TGT TAT GGC AAG ACA TTG GAG TAT AAC GAG TTC TAT CTG AGT CAA ATG ACC GAG TTA

           L   T   R   Y   G   D   V   E   E   V   W   L   D   G   A   K   G   E   G   -
Q7XUR3     CTC ACC AGG TAT GGA GAT GTG GAG GAA GTT TGG CTT GAT GGT GCA AAG GGA GAG GGC ---
           L   T   K   Y   G   E   I   K   E   V   W   L   D   G   A   K   G   D   G   E
Q8GW72     CTA ACA AAG TAT GGA GAG ATT AAG GAA GTT TGG TTA GAT GGA GCT AAA GGA GAC GGA GAG

           K   D   M   D   Y   M   F   D   A   W   F   A   L   I   H   Q   L   Q   Q   R
Q7XUR3     AAG GAC ATG GAT TAC ATG TTT GAT GCC TGG TTT GCA CTT ATC CAT CAG CTC CAG CAA AGG
           K   D   M   E   Y   F   F   D   T   W   F   S   L   I   H   Q   L   Q   P   K
Q8GW72     AAA GAT ATG GAA TAT TTC TTT GAT ACT TGG TTT AGT TTG ATC CAT CAG CTT CAG CCT AAA

           V   V   I   F   S   D   A   G   P   D   T   R   W   V   G   D   E   A   G   V
Q7XUR3     GTT GTC ATC TTC TCA GAT GCT GGA CCA GAT ACA AGA TGG GTA GGA GAT GAG GCA GGG GTC
           A   V   I   F   S   D   A   G   P   D   V   R   W   I   G   D   E   A   G   L
Q8GW72     GCT GTT ATA TTC TCT GAC GCT GGT CCT GAT GTC AGG TGG ATT GGT GAT GAA GCT GGT CTA

           A   G   Y   T   C   W   S   P   F   N   K   S   T   V   T   I   G   H   I   I
Q7XUR3     GCA GGT TAT ACT TGC TGG TCT CCT TTC AAT AAG AGC ACG GTG ACG ATT GGG CAC ATC ATT
           A   G   S   T   C   W   S   L   F   N   R   T   N   A   K   I   G   D   T   E
Q8GW72     GCT GGC TCT ACT TGC TGG TCA CTC TTT AAC AGA ACC AAT GCA AAG ATT GGT GAC ACT GAG

           P   E   Y   S   R   C   G   D   P   F   G   Q   D   W   V   P   A   E   C   D
```

Supplementary Figure 2. codon alignment used for estimating selection pressure. The sequences are from metazoans (A), fungi (B), and plants (C).
