# Supplementary material for: Origins and Evolution of the α-L-Fucosidases: From Bacteria to Metazoans
Source: Front Microbiol. 2019 Aug 27;10:1756. doi: 10.3389/fmicb.2019.01756 (PMC6718869; doi:10.3389/fmicb.2019.01756)
Supplement: Supplementary file 2 [file Table_1.DOCX]

Supplementary Table 1. Percent identity of the representative of α-l-fucosidases in the three subfamilies.

|  | *Homo sapiens* | *Bacteroidetes* | *Aspergillus* | *Streptomyces* | *Oryza sativa* | *Arenibacter* |
| --- | --- | --- | --- | --- | --- | --- |
| P04066 | 100 | 42.4 | 31.19 | 35.41 | 21.52 | 16.67 |
| A0A1F3LP43 | 42.4 | 100 | 31.97 | 34.56 | 22.56 | 16.89 |
| A0A146FA45 | 31.19 | 31.97 | 100 | 34.19 | 21.62 | 15.27 |
| A0A2G7AAY1 | 35.41 | 34.56 | 34.19 | 100 | 24.23 | 19.45 |
| Q7XUR3 | 21.52 | 22.56 | 21.62 | 24.23 | 100 | 29.78 |
| A0A318HTU7 | 16.67 | 16.89 | 15.27 | 19.45 | 29.78 | 100 |

The enzymes from the three subfamilies I, II, and III are shown in red, blue, and green, respectively, on the leftmost column. The UniProt IDs of the enzymes from the indicated organisms are shown.
